# Orthopedic Implant-Related Biofilm Pathophysiology: A Review of the Literature

**DOI:** 10.7759/cureus.15634

**Published:** 2021-06-14

**Authors:** Meletis Rozis, Dimitrios S Evangelopoulos, Spyros G Pneumaticos

**Affiliations:** 1 3rd Orthopaedic Department, National and Kapodistrian University of Athens, KAT Hospital, Athens, GRC

**Keywords:** biofilm formation, osteomyelitis pathophysiology

## Abstract

Orthopedic implant-related infections remain a major problem even nowadays. Bacterial resistance through biofilm formation, in addition to the limited treatment options available, has resulted in an increased effort to better understand pathophysiology mechanisms. We performed a review of the literature in order to identify major biofilm formation pathways through which possible treatment strategies could arise.

## Introduction and background

Orthopedic devices are in common practice worldwide for a wide number of procedures, including, among others, fracture treatment and total joint replacements (TJR). Implant-related infection, reported at rates of up to 5% [1], remains a major problem in orthopedic procedures. The fact that 76% of the patients with severe osteolysis (previously attributed to aseptic loosening) have been diagnosed with a biofilm by the use of our better diagnostic tools has highlighted its contribution to major orthopedic complications [2].

Biofilms complicate prosthetic joint implants at a rate of 1%-2% in primary joint replacement and in a percentage of 3%-5% in revision surgeries, even though rates as high as 20% have been referred [3]. The bone allografts, another common biomaterial in orthopedic procedures, have infection rates ranging from 4% to 12% [4]. The major bacteria responsible for periprosthetic infections are *Staphylococcus (S.) epidermidis* and *S. aureus* in 70% of all cases [5] while other bacteria are responsible for 22% of the infections [6]. 

We have performed a review of the literature about the critical mechanisms of biofilm formation and pathophysiology to provide a more in-depth understanding of this infectious entity.

## Review

Pathogenesis

Biofilms are organized communities of multiple species of bacteria and/or fungi embedded in an organic polymer matrix composed of extracellular deoxyribonucleic acid (DNA), proteins, and polysaccharides attached to a surface [7].

After the implantation, host cells and microbes compete with each other for an initial attachment to the implant surface, the so-called “race for surface,” as proposed by Gristina et al. [8]. In a short time after implant placement, a conditioning film of extracellular matrix (ECM) surrounds the biomaterial. This matrix, mainly composed of fibronectin, fibrinogen, albumin, vitronectin, collagen, and complement, is a lucrative substratum to which surrounding cells can adhere [9]. Host cells and planktonic bacteria compete with each other in that phase to first attach to the implant surface, a competence that will finally determine the fate of the implant itself.

The natural history of implant infection can be described in one of the following scenarios: (1) Initial inoculation of high virulent bacteria during surgery [10]; (2) Low virulence bacterial inoculation during surgery. In that case, there is a balance between the biofilm and the host immune system lost due to local factors; (3) Low virulence bacteria inoculated during surgery and expanding due to the host's uncompetitive immune system; (4) Bacteremia and hematogenous implant-related infection [11].

Biofilm formation stages

Whatever the infection mechanism, biofilm formation has four steps: (1) Adherence; (2) Accumulation; (3) Maturation; (4) Detachment.

Adherence

The key point in biofilm formation is the adherence of the planktonic bacteria to the biomaterial surface. This adherence has two phases, reversible and irreversible, with the first one being biologically less stable.

The initial, reversible phase is mainly determined by non-specific forces such as Van der Waals, electrostatic, acid-base, and hydrodynamic forces [12]. After being attracted by Van der Waals forces from a distance of 50-100 nm, hydrophobic interactions allow the bacteria to come closer to the surface (<1.5 nm) by water removal from the implant surface [13]. In a study by Gross et al., S. aureus mutants that lacked D-alanine esters in their membranes' teichoic acids showed minimized adhesion ability [14]. The fact that teichoic acids highly charge the cell membrane highlights the importance of electrostatic forces in the primary adhesion phase. The reversible phase ends with bonding the bacteria to the ECM components through the microbial surface components recognizing adhesive matrix molecules (MSCRAMMs) [15]. Those adhesins recognize molecules of the ECM, especially fibronectin (FN) and fibrinogen (Fg), with the first one having been proved to multiply the adherent ability of S. aureus due to fibronectin-binding protein A (FnBPA) and FnBPB binding proteins [16]. Herman-Bausier et al. studied the adherence of S. aureus and found that strong attachment is intermediated by homophilic interactions between the FnBPA and A -domains of adjacent cells [17]. In another study, Buck et al. underlined the importance of FnBPA and FnBPB, showing that alterations in their structures change the virulence potential of S. aureus [18]. In contrast, according to McCourt et al., mutant S. aureus of the USA300 lineage that had lost the ability to express FnBPA and FnBPB decreased their adherence ability [19].

Accumulation

After the bacteria-surface attachment, the next step is accumulation, a procedure referring to bacteria-to-bacteria adhesion. Bacteria produce a polysaccharide intercellular adhesin (PIA), which has a critical role in biofilm formation [20]. PIA is encoded by the icaA-B-C-D operon's genes and is negatively regulated by the icaR [21]. It is a homopolymer of β-1,6-linked N-acetylglucosamine residues that enhances the accumulation phase and helps them avoid phagocytosis from the host's immune system [22].

Even though PIA seems to have a critical role in the accumulation phase, not all biofilms are PIA-dependent. Besides, many bacterial species can accumulate due to several transmembrane proteins in a PIA-independent model [23].

S. epidermidis biofilms are studied in-depth due to the ica gene locus regulation. Even though the expression of the ica operon has been firmly associated with the ability of S. epidermidis to form a biofilm, such a biofilm includes PIA-producing colonies and PIA-negative ones [24]. Additionally, many studies have proved that S. aureus biofilms are not strictly based on PIA production [25], and other proteins, such as the accumulation-associated protein (Aap), biofilm-associated protein (Bap), and FnBPs [7,26], play a major role in these biofilms.

Maturation

Biofilms are rarely formed by one bacterial strain and are instead a balanced community of several species. This phase is characterized by the secretion of the biofilm glycocalyx, which structures its final morphology [27]. This glycocalyx acts as an anchoring system and provides stability and protection from the host's immune system. It is mainly anionic, composed of bacterial exopolysaccharides with long molecular chains of 0,5-2*10^6Da, and helps to entrap minerals and nutrients from the surrounding environment [28]. The glycocalyx is a crucial part of biofilm function. Colonies that produce it can host neighboring colonies with species that cannot do so inside the same biofilm [29]. These colonies communicate through interstitial voids (water channels) in which nutrients, signaling molecules, and extracellular DNA circulate [30]. Those signaling molecules and DNA exchange constitute the trans-colony communication (quorum sensing), which is the essential functional mechanism that gives the biofilm-embedded bacteria those special genotype and phenotype characteristics that make them unique in comparison to their homologous planktonic strains [31].

Detachment

After maturation, the embedded bacteria are placed in an organized structure inside the biofilm. Even though biofilm thickness ranges in-depth and despite the formed network for nutrients supply, these are not adequate for all the embedded bacteria [32]. This gradient of decreasing concentration in nutrients and oxygen promotes four distinct metabolic states inside a biofilm, ranging from cells under aerobic metabolism located near the surface to dormant and dead cells deeper in the biofilm. Rani et al. confirmed this observation after studying S. aureus biofilms, in which they described that cells closer to the surface created an anaerobic zone [33]. In contrast, two-thirds (2/3rd) of the biofilm were metabolically inactive. The bacteria located in the superficial active zone can detach from lack of nutrients and transport to adjacent areas creating new biofilms [34].

Biofilm resistance

Macrophages can phagocytose the planktonic bacteria but are unable to eliminate an organized biofilm; a phenomenon called "frustrated phagocytosis."

The bacteria that grow inside a biofilm have major differences from their planktonic forms. A lack of nutrients and other metabolic factors forces the embedded bacteria to decelerate their growing procedures, making them much more resistant to specific antibiotics. The extracellular polysaccharides offer additional protection, acting as a physical barrier to the host's immune system.

S. aureus biofilms have been well-studied for their resistance to antibiotics and the host's immune system. Those bacteria avoid phagocytosis by destroying local macrophages and polynuclear cells (PNC). The α-toxin is used by S. aureus to dissociate the macrophage membranes and thus kill them. Other pore-forming toxins with similar action are leukocidins. S aureus strains can produce up to five beta-barrel bicomponent leucocidins [35]. USA300 S. aureus' leukotoxins have been recognized, and these are LukSF, HlgAB and HlgCB, LukED, and LukAB [36]. Under the effect of those toxins, the macrophages become polarized and gain a poorly inflammatory phenotype, which, in turn, increases the biofilm resistance. At the same time, this is enhanced by the toll-like receptors' inability to detect the biofilm-embedded bacteria [37]. Other excreted proteins that make the bacteria less vulnerable when inside a biofilm have been discovered. S. aureus produces the staphylococcal complement inhibitor (SCIN), which blocks the classic complement pathways, and others like the chemotaxis inhibitory protein of staphylococci (CHIPS), the clumping factor A (ClfA), and the extracellular adherence protein (Eap) [34].

Nevertheless, probably the key role of biofilm general resistance is determined by genotype alteration. Bacteria produce molecules called autoinducers (AIs), creating a communication network in a population-dependent manner, the Quorum Sensing (QS). The AIs are different between gram-positive and gram-negative bacteria. The gram-negative bacteria mainly use the N-acyl homoserine lactones (AHLs) as signaling molecules [38] while molecules such as 2-heptyl-3-hydroxy-4-quinolone and diketopiperazines are involved [39]. Gram-positive bacteria communicate through oligopeptides [40]. The S. aureus biofilm is well-studied, and two communicating systems have been described, the accessory gene regulator system (arg) and the RAP/TRAP system [41]. The agr locus contains the AgrA, AgrB, AgrC, and AgrD genes (RNA2 transcript) and produces the auto-inducing peptide (AIP) [42]. S. aureus produces four allelic AIPs [43], which act as auto-feedback molecules for the AgrC/AgrA system and influence the transcription of RNA3 [44], which, in turn, regulates virulence factors like α-hemolysin [45]. Virulence factors translated by more than 200 genes are regulated by RNA3 and AgrA [46].

The RAP/TRAP QS system is ambiguous. Balaban et al. have speculated the RAP/TRAP system as an important QS regulatory pathway [47]. In this mechanism, an RNA3-activating protein (RAP) activates its' target protein (TRAP) which finally upregulates the agr system [48]. Shaw et al., on the other hand, found no evidence of RAP/TRAP pathway interference in the agr system and questioning its' significance in S. aureus QS and virulence [49]. The fact that QS regulates biofilm's resistance has gained attention as a possible new treatment strategy through QS interference, using hamamelitannin and vancomycin-releasing hydroxypropyl-β-cyclodextrin-functionalized cellulose gauzes [50].

## Conclusions

Infections remain a major problem in orthopedic procedures. There is an optimal difficulty not only in the treatment but in the diagnosis as well. The addition of laboratory tests and techniques, such as sonication, have altered our approach in common practice. The meaning, percentage, and importance of aseptic loosening are questioned nowadays, as the role of biofilms has emerged. Our better understanding of the cellular and molecular mechanisms that cause bone absorption, osteolysis, and, finally, implant failure further enhances biofilm warning. In the presence of micro-movement, mechanical-induced, and biofilm-induced osteolysis, the orthopedic implant loses its primary biomechanical characteristics, leading to increased complication rates. Collecting and reviewing those data, orthopedists should be more aware when opposing conditions of fracture-healing disturbance or joint prosthesis failure as a sub-clinical infection should be on top of the differential diagnosis. Disproportionately to the evolution of diagnostic tools, the treatment of biofilms is still in the initial stages. We have progressed in the last decade, but many studies are still needed to discover ways of biofilm destruction without implant removal.
